# Reassessment of Routine Midstream Culture in Diagnosis of Urinary Tract Infection

**DOI:** 10.1128/JCM.01452-18

**Published:** 2019-02-27

**Authors:** Sanchutha Sathiananthamoorthy, James Malone-Lee, Kiren Gill, Anna Tymon, Trang K. Nguyen, Shradha Gurung, Linda Collins, Anthony S. Kupelian, Sheela Swamy, Rajvinder Khasriya, David A. Spratt, Jennifer L. Rohn

**Affiliations:** a Centre for Nephrology, Division of Medicine, University College London, London, United Kingdom; b EDI Department of Microbial Diseases, University College London, London, United Kingdom; Marquette University

**Keywords:** 16S rRNA gene sequencing, lower urinary tract symptoms, midstream urine culture, mixed growth, urinary tract infection

## Abstract

Midstream urine (MSU) culture remains the gold standard diagnostic test for confirming urinary tract infection (UTI). We previously showed that patients with chronic lower urinary tract symptoms (LUTS) below the diagnostic cutoff on MSU culture may still harbor bacterial infection and that their antibiotic treatment was associated with symptom resolution. Here, we evaluated the results of the United Kingdom’s MSU culture in symptomatic patients and controls. Next, we compared the bacterial enrichment capabilities of the MSU culture with those of a 50-µl uncentrifuged culture, a 30-ml centrifuged sediment culture, and 16S rRNA gene sequencing. This study was conducted on urine specimens from 33 LUTS patients attending their first clinical appointment (mean age, 48.7 years; standard deviation [SD], 16.5 years), 30 LUTS patients on treatment (mean age, 47.8 years; SD, 16.5 years) whose symptoms had relapsed, and 29 asymptomatic controls (mean age, 40.7 years, SD, 15.7 years). We showed that the routine MSU culture, adopting the UK interpretation criteria tailored to acute UTI, failed to detect a variety of bacterial species, including recognized uropathogens. Moreover, the diagnostic MSU culture was unable to discriminate between patients and controls. In contrast, genomic analysis of urine enriched by centrifugation discriminated between the groups, generating a more accurate understanding of species richness. In conclusion, the United Kingdom’s MSU protocol misses a significant proportion of bacteria, which include recognized uropathogens, and may be unsuitable for excluding UTI in patients with LUTS.

## Abstract (precis)

Midstream urine (MSU) culture remains the gold standard diagnostic test for confirming urinary tract infection (UTI). We previously showed that patients with chronic lower urinary tract symptoms (LUTS) below the diagnostic cutoff on MSU culture may still harbor bacterial infection and that their antibiotic treatment was associated with symptom resolution.

## INTRODUCTION

An estimated 150 million to 250 million individuals worldwide develop urinary tract infection (UTI) every year, making it one of the most frequent types of human infections (1, 2). Midstream urine culture (MSU) is the gold standard test for its diagnosis. Hospital laboratories in the United Kingdom abide by the Standards for Microbiology Investigations (SMI B 41) protocol, issued and updated by Public Health England (PHE), which generally recommends a threshold of ≥10^5^ CFU/ml to confirm both uncomplicated and complicated UTI (3); this criterion is also adopted in many other countries but varies considerably. The 10^5^-CFU/ml threshold originates from a comparison conducted in the late 1950s of 74 pregnant female patients with clinically determined acute pyelonephritis and 337 asymptomatic women (4, 5).

Concerns regarding the suitability of this quantitative cutoff for patients with uncomplicated, acutely dysuric lower UTI were initially raised by Stamm and colleagues, and the evidence-based suggestion for lowering the colony count for this quantitative cutoff to 10^2^ CFU/ml was concluded to be the more appropriate alternative (6, 7). It is now recognized that culture, interpreted using a single quantitative threshold, may not be appropriate when used to detect different clinical manifestations of UTI (urethritis, cystitis, and pyelonephritis). This may be further complicated by a multitude of host and microbial factors (8, 9), making it increasingly likely that no single threshold is suitable for the detection of UTI in every clinical circumstance (10).

The current quantitative MSU threshold is problematic for other reasons. First, bacterial strains are known to vary in virulence (11), meaning that some strains might generate disease even at lower concentrations. Second, the concentration of bacteria in urine can vary widely depending on fluid intake before MSU sampling; the concentration in a dilute specimen might fall below the diagnostic cutoff as a result. Third, recognized uropathogens, including Escherichia coli and Enterococcus faecalis, adhere to host cells and are also known to invade them to form intracellular colonies (12–14). Thus, urinary supernatant may be a poor substrate for recovering sequestered organisms. However, it is also known that an innate immune response to UTI exacerbates the shedding of urothelial cells into urine (15–19).

An additional problem associated with standard MSU interpretation in the United Kingdom is the dismissal of mixed-growth cultures by default. Conventionally, mixed-growth cultures are assumed to reflect contamination by organisms recognized to colonize the healthy periurethral, vaginal, and perianal regions (5). Although some laboratories electively consider the growth of up to three organisms each at >10^5^ CFU/ml to suggest polymicrobial infection or may proceed with a repeat assessment, many disregard mixed cultures altogether (3). However, we and others have shown that polymicrobial infection is common in patients with lower urinary tract symptoms (LUTS) (13, 20–22). In addition, polymicrobial urosepsis has been reported in patients who demonstrated identical, mixed isolates from blood and urine specimens (23, 24). Another study reported that E. coli, a predominant uropathogen, manifested greater invasive properties when isolated from a polymicrobial culture than when isolated as pure growth (25). This finding is supported by recent work showing that E. faecalis can bolster E. coli by exporting the nutrient l-ornithine (26).

Few studies have analyzed the urinary microbial composition by laboratory outcome (i.e., negative, mixed, positive cultures), and the majority of such work has assessed culture techniques adopted in U.S. laboratories (6, 27–30). Given the limitations of culture-based microbial detection (31–33), characterization of microbial communities in health and disease is now becoming increasingly dependent on approaches, such as DNA-based identification, which do not require growth under particular culture conditions (34). Using metagenomics, the urinary microbiota in patients with neurogenic bladder dysfunction (35), overactive bladder (36), urgency urinary incontinence (UUI) (28), stress urinary incontinence (SUI) (37), and uncomplicated UTI (38) have been described. Some studies have also included comparisons to asymptomatic individuals (28, 33, 35, 36, 39–41).

A critical microbiological evaluation of the urine culture protocol adopted in the United Kingdom is long overdue. Here, we chose to study MSU culture performance in patients describing LUTS, including those who fall short of a positive culture and those demonstrating mixed growth, since these are generally deemed not to have a *bona fide* infection. This assertion hinges on standard culture-based diagnosis, and emerging data (13, 28, 42) suggest that such patients harbor chronic infection. Our aim was to evaluate the MSU culture in symptomatic patients and asymptomatic controls and assess its performance using both culture and molecular approaches with and without specimen enrichment.

## MATERIALS AND METHODS

### Subject recruitment and clinical assessment.

**FIG 1:**
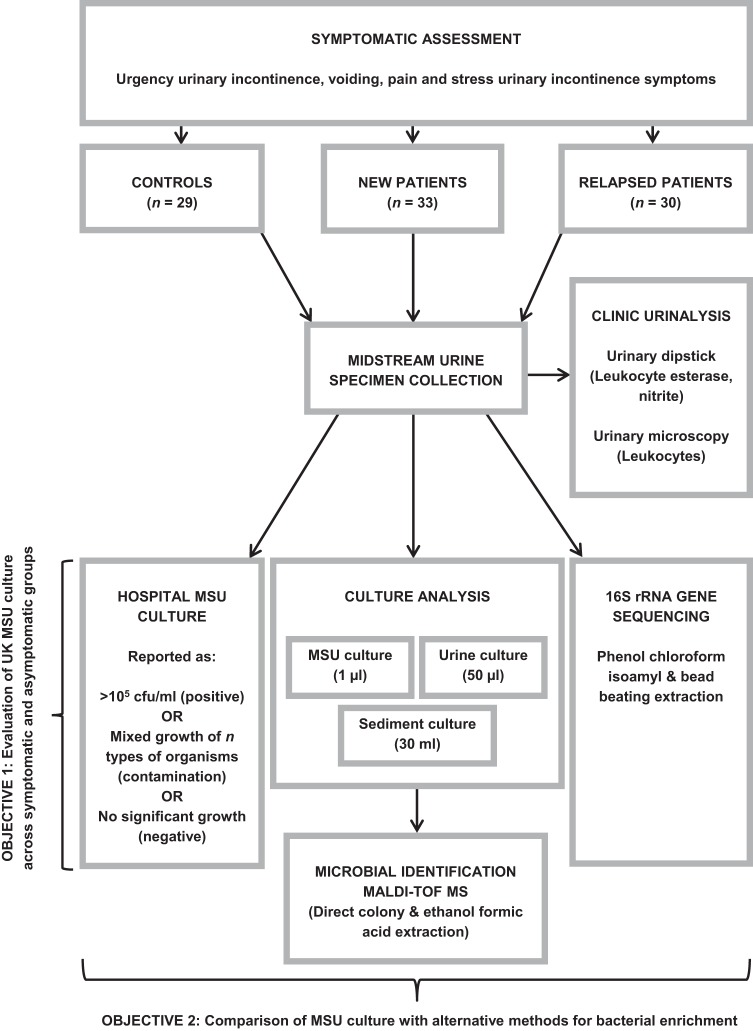
Experimental work flow of the study. Abbreviation: MALDI-TOF MS, matrix-assisted laser desorption ionization–time of flight mass spectrometry.

This study obtained ethical approval from the East London & the City Research Ethics Committee, London, United Kingdom. Adult men and women aged ≥18 years with LUTS were eligible for study inclusion. Pregnant subjects were not included in the study. Since urine cultures are relied upon for diagnosis at initial presentation and during relapse, two separate symptomatic patient groups were recruited and assessed. Patients attending their first appointment at the Whittington Hospital Lower Urinary Tract Symptoms (LUTS) Clinic (referred to in this study as “new patients”), who were not on antibiotic treatment and who had not taken antibiotics in the preceding 4 weeks, were evaluated by the clinician present and inducted into the first patient group. Patients attending a follow-up consultation with symptomatic recurrence, relapse, or no response to initial antimicrobial treatment (referred to as “relapsed patients”) were identified following clinical assessment to form the second patient group. Asymptomatic controls aged ≥18 years of either sex who had no urological complications and who were not on antibiotic treatment were recruited from departmental staff, students, and the general population. All patients and controls provided written consent prior to study inclusion. Figure 1 presents a work flow of all experimental procedures conducted on each subject. Clinicians overseeing the care of new and relapsed patients were blind to all urinalysis results (i.e., urinary dipstick, microscopy, and MSU culture test results). For all downstream analyses, the culture plates were coded with a four-digit study number and processed separately in large batches. The data for each technique were inputted separately, and comparisons did not take place until all of the data had been entered and the code broken.

Symptoms of urgency urinary incontinence (UUI), voiding dysfunction, pain, and stress urinary incontinence (SUI) were recorded using a validated questionnaire with questions requiring a yes-or-no response (43). The context-related symptoms were summed within each of the four symptom groups. Patient and control urine specimens were obtained using the clean-catch MSU method and anonymized with a four-digit study number. Subjects were carefully instructed in the collection technique. Each urine specimen was analyzed using urinary dipsticks and an automated Clinitek Status analyzer (Siemens Healthcare, Germany) for leukocyte esterase and nitrites. Microscopic leukocyte counts were determined using a Neubauer counting chamber, loaded with 10 µl of fresh urine. Each prepared specimen was examined by microscopy (magnification, ×200) using an Olympus CX41 light microscope (Olympus, UK).

### National routine diagnostic screening.

An aliquot of each MSU specimen was submitted to the Whittington Hospital Microbiology Laboratory, London, United Kingdom, for routine culture. Urine specimens were stored at 4°C until they were transported to the processing laboratory. These samples took approximately 60 min to arrive and were cultured either immediately on the same day or on the next day following overnight storage at 4°C, which reflected usual practice. The protocol involved inoculating ChromID CPS (now ChromID CPS Elite) chromogenic culture medium (bioMérieux, France) with 1 μl of uncentrifuged urine, which was then placed in an ordinary incubator at 37°C. Microbial colonies were identified using the manufacturer’s color criteria. A count of ≥10^5^ CFU/ml for one organism was interpreted as a significant result. Cultures with a colony count below this threshold were reported as “no significant growth.” MSU cultures with more than one organism were reported as “mixed growth of *n* types of organisms.”

### Extended culture-based analysis.

Urine samples were stored at 4°C for 0.5 to 4.0 h before transportation to the research laboratory (University College London, London, UK), where they were processed immediately. An identical MSU culture was performed on an aliquot of the same urine specimen. Simultaneously with this culture, 50 µl of uncentrifuged urine was plated on chromogenic agar. From the remaining aliquots of each specimen, 30 ml was centrifuged at 1,400 × *g* for 10 min. The sediment was resuspended in 400 µl of sterilized phosphate-buffered saline (PBS) solution (Life Technologies, UK). Tenfold serial dilutions were performed using PBS to reveal the presence of morphologically small-sized colonies where the growth of other organisms dominated. All cultures were incubated aerobically at 37°C for 18 to 24 h.

### Identification of cultured isolates.

Microbial isolates were identified using matrix-assisted laser desorption ionization–time of flight mass spectrometry (MALDI-TOF MS) and a MicroFlex LT mass spectrometer (Bruker Daltonics, USA). In the first instance, the direct colony plate method was employed for identification, and each cultured isolate was inoculated twice onto the target plate. The target plate was left to air dry before applying 1 µl of matrix solution (Bruker Daltonics, USA) consisting of alpha-cyano-4-hydroxycinnamic acid dissolved in 50% acetonitrile and 2.5% trifluoroacetic acid. The air-dried target plate was inserted into the mass spectrometer, and time was given for the high vacuum to be restored. Using the MALDI Biotyper (version 3.0) software program (Bruker Daltonics, USA), the isolate identifiers were recorded onto the program and the Bruker Taxonomy library was selected for the analysis. In the event of an unsuccessful identification, an ethanol-formic acid extraction protocol was adopted to assist with microbial cell wall disruption and release of ribosomal proteins (44). A heavy suspension of the unidentified isolate was made in 300 µl of sterile distilled water. Following the addition of the absolute ethanol (900 µl) microbial suspension, samples were spun down at 20,000 × *g* for 2 min. The resulting ethanol was discarded and the centrifugation step was repeated. The residual ethanol was removed, and the resulting pellet was left to air dry at room temperature for 5 min. The deposit was resuspended in 70% formic acid (20 to 50 µl). An equal volume of acetonitrile was added to this mixture, followed by centrifugation at 20,000 × *g* for 2 min. The supernatant was then applied onto a sterilized target plate twice and left to air dry, before resuming with the direct approach protocol from the point of adding matrix solution to each dried spot.

### 16S rRNA gene sequencing of urine.

Prior to extraction, urine samples for DNA sequencing were stored at −80°C. Genomic DNA was extracted from each specimen using an approach adapted from a previously reported phenol-chloroform-isoamyl alcohol and bead-beating method (45). Specimens were spun down, using a precooled centrifuge, at 18,000 × *g* for 5 min at 4°C. The cell pellets were resuspended in extraction buffer (500 µl) consisting of 120 mM potassium phosphate (K_2_PO_4_) buffer (pH 8.0) with 5% cetyltrimethylammonium bromide (CTAB) (Sigma-Aldrich, USA) in 0.7 M NaCl. The specimen tubes were vortexed, and the contents were transferred into presterilized 2-ml tubes containing zirconia/silica beads (diameter, 0.1 mm). Phenol-chloroform-isoamyl alcohol (PCI; 25:24:1; 500 µl; Invitrogen, USA) was added to each sample, and the mixture was kept on ice. The samples were homogenized using a RiboLyser apparatus (Hybaid, Germany) for 30 s at 5.5 m/s and spun down at 18,000 × *g* for 15 min. Chloroform-isoamyl alcohol (CI; Invitrogen, USA) was added at a 1:1 ratio to the extracted layer of DNA supernatant. After centrifugation at 18,000 × *g* for 2 min at 4°C, the DNA of each sample was precipitated by adding 30% polyethylene glycol 8000 (PEG 8000; Sigma-Aldrich, USA) at a 2:1 ratio.

PCR was performed to amplify the V5-V7 hypervariable regions of the 16S rRNA gene using the barcoded primers 785F (5′-GGATTAGATACCCBRGTAGTC-3′) and 1175R (5′-ACGTCRTCCCCDCCTTCCTC-3′) (see Table S1 in the supplemental material) (Sigma, UK). Each 25-µl sample reaction mixture contained 0.125 µl Moltaq DNA polymerase (0.025 µM; Molzym, VH Bio Ltd., UK), 2.5 µl of Molzym buffer (1×) (Molzym, VH Bio Ltd., UK), 0.5 mM MgCl_2_, 0.2 mM deoxynucleoside triphosphates (Bioline, UK), 1 µl of the forward and reverse primers (0.4 µM), and PCR water (Molzym, VH Bio Ltd., UK). The PCR stages involved an initial denaturation step at 95°C for 5 min and subsequent amplification for 30 cycles at 94°C for 30 s (denaturation), 54°C for 40 s (annealing), and 72°C for 60 s (elongation), followed by 72°C for 10 min and a final hold of 4°C. All sample reactions were performed in duplex. The DNA concentrations were determined using a Qubit high-sensitivity DNA assay kit (Life Technologies, UK) and a Qubit (version 2.0) fluorometer (Life Technologies, UK). The amplicons were purified using Agencourt AMPure XP-PCR magnetic particles (Beckman Coulter, USA) and combined in equimolar ratios using elution buffer (Qiagen, UK) to generate three pooled DNA libraries (libraries 1, 2, and 3) for pyrosequencing on a MiSeq desktop sequencer (Illumina Inc., USA), using the version 2 reagent kit (Illumina Inc., USA). Since library 3 contained samples with the lowest DNA yields, the purification step was repeated for this pooled library in an attempt to improve on the final DNA concentration.

Our own customized and commercial mock communities were incorporated into DNA libraries 1 and 2 to verify the DNA sequencing protocol (Fig. S1).

### Statistical analysis.

All descriptive and inferential statistics were generated using SPSS (version 25.0) software (IBM, USA). Demographic data, age, gender, 24-h urinary frequency, nocturia, validated LUTS scores, and log-transformed leukocyte counts were compared across the groups using nonparametric tests. In all cases except age, homogeneity of variance was not shown, so Mood’s median test was used instead of the Kruskal-Wallis test. Hospital MSU culture data (with the outcomes “negative,” “mixed growth,” and “positive culture”) were compared across the study groups using the chi-square test or Fisher’s exact test.

The 16S rRNA data were analyzed using QIIME (Quantitative Insights into Microbial Ecology, version 1.8; qiime.org) software (46). The raw reads were demultiplexed and assigned to samples using barcoded sequences. After demultiplexing, the paired-end data were joined to obtain a single FASTQ file for each sample. These sequences were then clustered into operational taxonomic units (OTUs) using an open-reference OTU picking strategy. The OTU clusters were assigned to the Greengenes Reference Database (http://greengenes.lbl.gov) (47) based on 97% sequence similarity (46). OTUs were then filtered to remove the chimeric sequences (UCHIME) (48), and taxonomy was assigned using the Ribosomal Database Project (RDP) classifier (49). Core diversity analyses based on study group and sample type were performed.

## RESULTS

### Clinical characteristics of the patient cohorts providing samples.

As previous work suggested that the gold standard MSU culture was not an optimal diagnostic tool to detect infection in LUTS patients, we set out to directly compare it with two alternative culture techniques. We collected clean-catch MSU specimens from 33 untreated new patients (mean age, 48.7 years; standard deviation [SD], 16.5 years) and 30 patients experiencing a symptomatic relapse (mean age, 47.8 years; SD, 16.5 years). Since urine cultures are relied upon for diagnosis at initial presentation and during relapse, these two patient groups were recruited and assessed separately. For comparison, we also recruited 29 asymptomatic controls (mean age, 40.7 years; SD, 15.7 years). Table 1 details the clinical characteristics of each study cohort. Statistically similar age distributions were observed for all three study groups (χ^2^ = 4.4, degrees of freedom [df] = 2, *P* = 0.113). The majority of patients were female, which reflected the well-known demographics of UTI.

**TABLE 1:** Clinical characteristics of each study group^*e*^

Characteristic	Value(s) for:			*P* value
	New patients (*n* = 33)	Relapsed patients (*n* = 30)	Controls (*n* = 29)	
Demographics				
No. (%) patients by sex				
Female	32 (97.0)	27 (90.0)	26 (89.7)	
Male	1 (3.0)	3 (10.0)	3 (10.3)	
Age (yr)				
Mean (SD)	48.7 (16.5)	47.8 (16.5)	40.7 (15.7)	0.113^*a*^
Range (yr)	18–77	24–78	20–76	
Urinary patterns (24 h)				
Frequency				**<0.05**^*b*^
Mean	8.8	8.5	5.9	
SD	5.1	3.7	1.5	
Median (95% CI)	6.5 (5.5–10.5)	8.0 (6.5–9.5)	5.5 (5.5–6.5)	
Nocturia				**<0.05**^*b*^
Mean	1.7	1.6	0.3	
SD	1.7	1.9	0.4	
Median (95% CI)	1.5 (0.5–2.0)	1.0 (0.0–2.5)	0.0 (0.0–0.5)	
No. (%) of patients with the following symptoms^*d*^:				
UUI	25 (75.8)	12 (40.0)	0 (0.0)	**<0.05**^*c*^
Pain	24 (72.7)	26 (86.7)	0 (0.0)	0.172^*c*^
Voiding	29 (87.9)	21 (70.0)	0 (0.0)	0.080^*c*^
SUI	17 (51.5)	2 (6.7)	0 (0.0)	**<0.001**^*c*^
No. of symptoms				
UUI symptoms				**<0.001**^*b*^
Mean	3.4	1.4	0.0	
SD	2.9	2.2	0.0	
Median (95% CI)	3.0 (1.0–4.0)	0.0 (0.0–2.0)	0.0 (0.0–0.0)	
Pain symptoms				**<0.001**^*b*^
Mean	3.1	3.6	0.0	
SD	2.7	2.9	0.0	
Median (95% CI)	3.0 (1.0-4.0)	3.0 (2.0–4.0)	0.0 (0.0–0.0)	
Voiding symptoms				**<0.001**^*b*^
Mean	4.1	3.6	0.0	
SD	2.8	2.8	0.0	
Median (95% CI)	4.0 (2.0–6.0)	4.0 (2.0–5.0)	0.0 (0.0–0.0)	
SUI symptoms				**<0.001**^*b*^
Mean	0.7	0.3	0.0	
SD	0.8	1.0	0.0	
Median (95% CI)	1.0 (0.0–1.0)	0.0 (0.0–0.0)	0.0 (0.0–0.0)	
Total symptom score				**<0.001**^*b*^
Mean	11.3	8.9	0.0	
SD	5.9	5.2	0.0	
Median (95% CI)	12.0 (9.0–14.0)	9.0 (6.0–11.0)	0.0 (0.0–0.0)	

a Kruskal-Wallis test.

b Mood’s median test.

c Chi-square test.

d Percentages are those within each group. Statistical comparisons are between the new patient and relapsed patient groups only. Symptoms were ascertained from a yes-or-no response to questions on a questionnaire.

e Abbreviations: CI, confidence interval; SUI, stress urinary incontinence; UUI, urgency urinary incontinence. Statistically significant differences are indicated by bold font.

First, we inspected the differences between the patient and control cohorts. None of the controls reported any LUTS, thus differing significantly from both patient cohorts studied. A review of the symptomatic history of new patients (*n* = 30, the duration of symptoms was not recorded for three patients) revealed that 1 patient (3.0%) had experienced symptoms for <1 year, 16 patients (48.5%) had experienced symptoms for 1 to 4 years, 9 patients (27.3%) had experienced symptoms for 5 to 10 years, and 4 patients (12.1%) had experienced symptoms for ≥10 years. The median number of daytime and nighttime urinary episodes differed across the three groups, with pairwise *post hoc* comparisons identifying a higher median frequency for new patients and relapsed patients than for the controls (Table 1). We proceeded to examine the symptomatic differences between new and relapsed patients. Comparisons of symptom presentation between patient groups revealed that the number of new patients reporting UUI and SUI symptoms was significantly higher than that of relapsed patients. The proportions of new and relapsed patients that reported voiding symptoms and pain symptoms were statistically similar (Table 1).

In addition to observing the presence and absence of symptoms, we also calculated a symptom score to determine the magnitude of LUTS within each patient group. The number of symptoms was summed for each of the four categories to provide a score and compared across the groups using Mood’s median test. The median symptom scores for UUI, SUI, voiding, and pain symptom categories differed across the three study groups. *Post hoc* analysis revealed higher median UUI, SUI, and voiding scores for new patients than relapsed patients. However, both patient cohorts had similar pain scores and total symptom scores (Table 1). Statistical comparison of the respective frequencies for each assessed LUTS between new and relapsed patient groups are provided within the supplemental material (Table S2).

### Urinary microscopy and dipstick analysis.

We performed urinary leukocyte counts to determine whether patient urine manifested evidence of infection independent of the bacterial assessment. As shown in Table 2, there was a significant difference in the log_10_ leukocyte counts among the three study groups (χ^2^ = 6.2, df = 2, *P* < 0.05). *Post hoc* analysis using Bonferroni’s correction identified significant differences between controls and new patients and between controls and relapsed patients. No significant difference in the log_10_ leukocyte counts was identified between new patients and relapsed patients.

**TABLE 2:** Descriptive measures of in-house (clinic) microscopic leukocyte counts and reported routine hospital MSU culture results for each study group^*a*^

Diagnostic test	Value^*d*^ for:		
	New patients (*n* = 33)	Relapsed patients (*n* = 30)	Controls (*n* = 29)
Clinic microscopic leukocyte count (log_10_ no. of wbc/µl)			
Mean ± SD (95% CI)	1.7 ± 0.9 (0.3–1.0)	0.7 ± 1.0 (0.4–1.1)	0.2 ± 0.4 (0.1–0.3)
Median (95% CI)	0.3 (0.0–0.3)	0.5 (0.0–0.8)	0.0 (0.0–0.8)
Frequency (% of group)			
0 wbc/µl	15 (45.5)	12 (40.0)	20 (69.0)
1–9 wbc/µl	11 (33.3)	11 (36.7)	8 (27.6)
≥10 wbc/µl	7 (21.2)	7 (23.3)	1 (3.4)
No. (%) of patients with the following hospital midstream urine culture findings:			
No significant growth^*b*^	21 (63.6)	23 (76.7)	22 (75.9)
Mixed growth^*c*^ of two organisms	3 (9.1)	2 (6.7)	4 (13.8)
Mixed growth of three organisms	4 (12.1)	2 (6.7)	1 (3.4)
>10^5^ CFU/ml of one organism	4 (12.1)	2 (6.7)	2 (6.9)

a Abbreviations: wbc, white blood cell; CI, confidence interval.

b Less than 10^5^ organisms/ml or the growth of ≥2 organisms at <100 colonies.

c Greater than 100 colonies.

d Midstream urine culture results were untraceable for new patients (*n* = 1) and relapsed patients (*n* = 1).

Analysis of the urinary dipstick results showed that 13 (39.4%) new patients, 11 (36.7%) relapsed patients, and 6 (20.7%) controls tested positive for leukocyte esterase, with only 1 (3.0%) new patient testing positive for urinary nitrite. The results were unsurprising, as previous work suggested that the dipstick is not sensitive enough to diagnose infection (leukocyte esterase = 46 to 66%, nitrite = 6 to 18%), particularly in patients with nonacute symptoms of UTI (50–52).

Although LUTS can be driven by noninfectious causes, the association between patient symptoms and leukocyte recruitment demonstrates urinary tract inflammation. This is critical if an infective etiology for LUTS is being considered.

### Hospital MSU culture results.

Next, we determined the relationship between patient symptoms and their standard MSU results. Table 2 summarizes the data, in which the majority of new patients, relapsed patients, and controls were reported to have a negative MSU culture. Six (9.5%) symptomatic patients showed a positive culture result, as did two (6.9%) controls. A comparison of the hospital MSU culture outcomes revealed that the MSU culture was unable to discriminate between the three study groups (χ^2^ = 1.7, df = 4, *P* = 0.787). The hospital reports showed that cultures for all four new patients with positive cultures grew E. coli, whereas *Enterococcus* (*n* = 1) and a coliform belonging to the *Klebsiella/Enterobacter/Serratia* group (*n* = 1) were reported for the positive MSU cultures for relapse patients. *Proteus* (*n* = 1) and Streptococcus agalactiae (*n* = 1) were cultivated from the controls.

Further pairwise statistical comparisons of the number of positive and negative MSU cultures using Fisher’s exact test were performed. For this particular analysis, cultures with mixed growth and no significant growth were under the category of “negative,” as both results are traditionally dismissed as insignificant. This analysis indicated that even with the exclusion of the relapsed group, the MSU culture was still unable to discriminate between asymptomatic controls and new patients (χ^2^ = 0.539, df = 1, *P* = 0.674). The same analysis for relapsed patients and controls also showed no significant difference (χ^2^ = 0.0, df = 1, *P* = 1.0). Likewise, comparison of new patients and relapsed patients showed no difference (χ^2^ = 0.539, df = 1, *P* = 0.674). These results further highlight the inability of the MSU culture to discriminate between the three study groups.

Since the hospital laboratory did not report the microbial composites of mixed-growth and negative MSU cultures, we inspected the 1-µl-loop MSU cultures that were replicated in our own research laboratories, subjecting colonies grown on chromogenic agar to MALDI-TOF MS analysis for identification. The pooled percentage frequencies of organisms identified from the polymicrobial MSU cultures of new patients (*n* = 7), relapsed patients (*n* = 4), and controls (*n* = 5) are shown in Fig. S2. No colonies on the research laboratory MSU culture were observed for 17 (58.6%) controls, 12 (36.4%) new patients, and 13 (43.3%) relapsed patients. While 19 isolates were grown from 12 asymptomatic control MSU cultures, 39 isolates were identified from 21 new patient cultures and 25 isolates were grown from 17 relapsed patient cultures. With this replicated diagnostic culture, *Enterococcus* and *Escherichia* were predominantly cultured from new patients and relapsed patients, whereas *Streptococcus* and *Staphylococcus* were most frequently isolated from the controls. Within this sample size, Fisher’s exact test confirmed a significantly higher frequency of *Escherichia* (χ^2^ = 5.4, df = 1, *P < *0.05) and *Enterococcus* (χ^2^ = 8.9, df = 1, *P < *0.05) isolated from cultures for new patients than from those for the controls. The relapsing patient group was also observed to produce a significantly higher number of *Enterococcus* isolates than the control group (χ^2^ = 6.4, df = 1, *P < *0.05). In summary, the quantitative MSU had no discriminatory diagnostic power, but the microbial communities isolated in the process showed some differences between the patients and the controls.

### Comparison of culture techniques.

**FIG 2:**
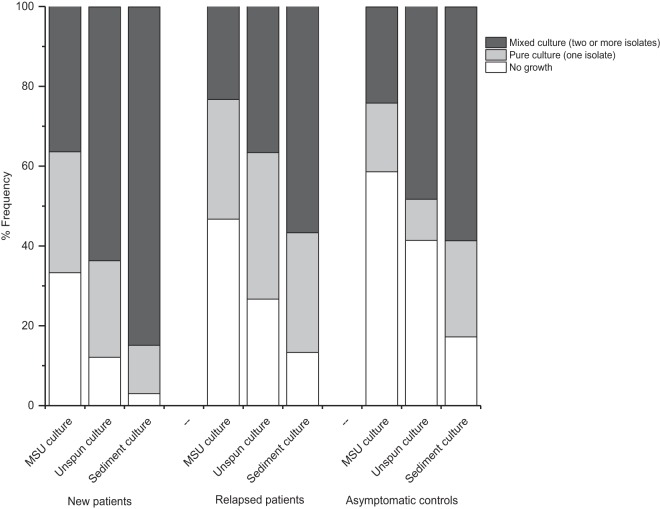
Composition types (no growth, pure and mixed cultures) achieved with three different techniques (MSU culture [1 µl], unspun culture [50 µl], and sediment culture [30 ml]) across study groups.

We compared the discriminatory power of the hospital MSU culture with that of other methods, specifically, plating a larger volume of uncentrifuged urine and plating samples enriched by centrifugation. As shown in Fig. 2, microbial growth was categorized as “no growth,” “one isolate,” and “two or more isolates” for comparison. Of note, as the amount of bacterial input increased (from 1 µl supernatant to 50 µl supernatant of centrifuged sediment), regardless of the patient group, more polymicrobial growths were revealed. Using this alternative categorization, the MSU culture replicated within our research laboratories was still unable to discriminate between the three study groups (χ^2^ = 4.6, df = 4, *P* = 0.326). Additionally, culturing more supernatant (50 µl) or the sediment did not improve differentiation across the three cohorts (χ^2^ = 11.7, df = 4, *P* = 0.02 [no significant differences were identified with *post hoc* analysis] and χ^2^ = 8.4, df = 4, *P* = 0.078, respectively), likely because, as we and others have reported, healthy bladders also harbor polymicrobial growths. These results suggest that quantitative microbiology is not an adequate diagnostic tool for patients experiencing LUTS.

### Patient and control urinary bacterial communities.

**FIG 3:**
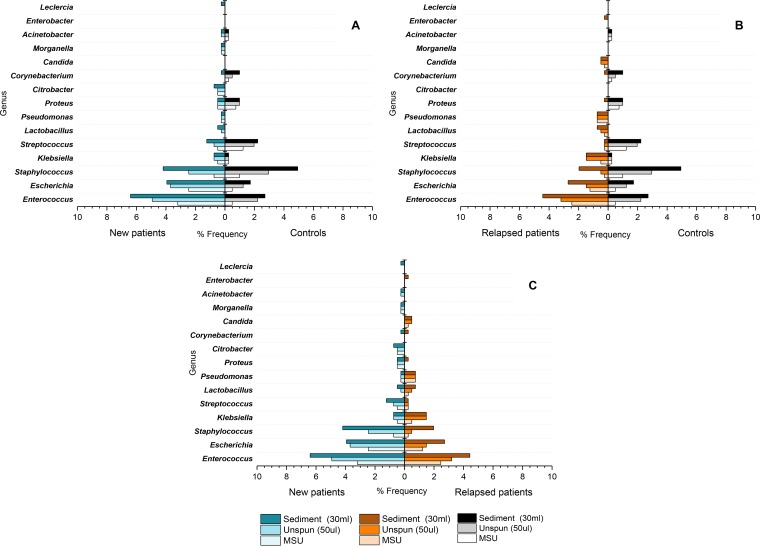
Pairwise comparisons of the percent frequencies of organisms identified from the urine specimens of new patients (*n* = 33), relapsed patients (*n* = 30), and controls (*n* = 29) using three different culture techniques: routine midstream urine (MSU) culture, unspun culture of a 50-µl sample volume, and a spun sediment culture of a 30-ml sample volume. (A) New patients versus controls; (B) relapsed patients versus asymptomatic controls; (C) new patients versus relapsed patients. Data are presented as the percentages of the total number of isolates identified.

Figure 3 illustrates pairwise study group comparisons of the genera and the respective percentage frequencies identified on the MSU culture (reproduced in-house), 50-µl urine unspun culture, and 30-ml sediment cultures (see Fig. S3 for the species-level characterization across study groups). As seen with the MSU culture results, differences were noted among the various groups on sediment culture. While 15 different genera were grown from symptomatic patient sediment cultures (*n* = 13 from new patients, *n* = 11 from relapsed patients), 8 genera were isolated from control sediment cultures. Seven genera were shared by both patient and control groups; these were *Corynebacterium*, *Enterococcus*, *Escherichia*, *Klebsiella*, *Proteus*, *Staphylococcus*, and *Streptococcus*. Organisms that were cultivated from patient samples but that were not isolated from control specimens were *Candida*, *Citrobacter*, *Enterobacter*, *Lactobacillus*, *Leclercia*, *Morganella*, and *Pseudomonas*. In both symptomatic patient groups, *Enterococcus*, *Staphylococcus*, and *Escherichia* were the most abundant organisms. In the asymptomatic controls, *Staphylococcus*, *Enterococcus*, and *Streptococcus* were the most frequently isolated. However, *Staphylococcus* (χ^2^ = 3.7, df = 1, *P* = 0.064) and *Streptococcus* (χ^2^ = 2.4, df = 1, *P* = 0.2) were present at statistically similar frequencies in patients. Fisher’s exact test revealed a significantly higher frequency of *Enterococcus* isolates cultivated from the new patients than from the asymptomatic controls (χ^2^ = 6.2, df = 1, *P < *0.05). No significant difference was observed with any other genus between new patients and controls. Comparison of the frequencies between the control and relapsed patient groups using Fisher’s exact test revealed a significantly higher frequency of *Staphylococcus* (χ^2^ = 6.9, df = 1, *P < *0.05) and *Streptococcus* (χ^2^ = 7.0, df = 1, *P < *0.05) in controls than in relapsed patients. No significant differences in the number of genera were identified between the new and the relapsed patient groups.

We went on to analyze urinary bacteria in unprocessed urine (1 ml, uncentrifuged) versus 30 ml of urine enriched by centrifugation in the new patient versus control study groups using next-generation sequencing based on the rRNA gene. The percentage of identified sequencing reads or reads that passed filter (PF) (percentage of bases with a quality score of ≥30 [%Q≥30]) for loaded DNA libraries 1, 2, and 3 were (i) 77.9% (10,122,606 PF reads), (ii) 80.0% (14,910,030 PF reads), and (iii) 68.3% (10,129,563 PF reads), respectively. Bacterial DNA was detected in the urine samples from 32 (97.0%) of 33 recruited new patients (30 uncentrifuged urine samples and 30 centrifuged samples) and 26 (89.7%) of 29 control patients (22 uncentrifuged samples and 23 centrifuged samples). Samples that yielded low DNA concentrations were salvaged with an extended protocol. Beyond this, bacterial DNA was undetectable in one new patient and three controls.

**FIG 4:**
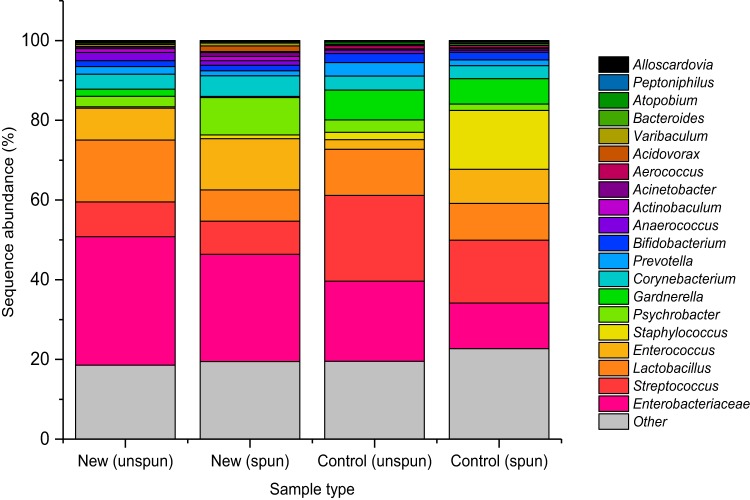
Percentage sequence abundances of the 20 most abundant taxa detected in both new patient and control cohorts when categorized by sample type.

Figure 4 presents the relative abundance of the 20 most abundant taxa identified from the uncentrifuged and centrifuged urine samples for new patients and controls (see Table S3 for hierarchical classifications). The 20 most abundant taxa made up 81.0% of new patient sequences and 79.0% of control sequences. The most abundant taxa of the uncentrifuged urinary bacterial community of new patients from highest to lowest were *Enterobacteriaceae* (32.3%), followed by *Lactobacillus* (15.5%), *Streptococcus* (8.7%), and *Enterococcus* (8.0%), whereas the most abundant taxa of the new patient urinary microbial community represented by centrifuged samples were *Enterobacteriaceae* (26.9%), *Enterococcus* (12.8%), *Psychrobacter* (9.3%), and *Streptococcus* (8.3%). The most abundant taxa identified from uncentrifuged control samples were *Streptococcus* (21.5%), *Enterobacteriaceae* (20.1%), *Lactobacillus* (11.6%), and *Gardnerella* (7.5%). In contrast, the most abundant taxa identified from centrifuged control urine samples were *Streptococcus* (15.8%), *Staphylococcus* (14.8%), *Enterobacteriaceae* (11.5%), and *Lactobacillus* (9.2%). From these data, a decrease in the overall abundance of *Lactobacillus* (15.5% uncentrifuged, 7.8% centrifuged) and an increase in the abundance of *Enterococcus* (8.0% uncentrifuged, 12.8% centrifuged) were observed with the new patient centrifuged urinary community compared with the abundances observed with the new patient uncentrifuged community. On the other hand, the control centrifuged community showed an increase in the overall abundance of *Staphylococcus* (1.9% uncentrifuged, 14.8% centrifuged) compared with that in the control uncentrifuged urinary community. Centrifugation of samples therefore has a strong influence on species recovery. Moreover, this approach also highlights differences in microbial composition between patients and controls (similar to what was seen in our sediment culture data [Fig. 3]).

Table 3 presents the richness and diversity measures of new patient and control urinary tract bacterial communities. Richness was assessed by comparing the mean number of OTUs and mean chao1 estimator values using Welch’s two-sample *t* test. Diversity was assessed by comparing the mean Shannon index and mean inverse Simpson’s index using Welch’s two-sample *t* test. Pairwise comparisons indicated no significant difference in the mean number of observed OTUs and the chao1 estimator between patient and control uncentrifuged samples, centrifuged samples, and both uncentrifuged and centrifuged samples combined. Additionally, no significant difference in the mean Shannon’s index and inverse Simpson’s index was observed between the patient and the control communities.

**TABLE 3:** Richness and diversity measures determined for new patients and controls

Metric	New patients			Controls		
	Unspun (*n* = 30)	Spun (*n* = 30)	Both (*n* = 60)	Unspun (*n* = 22)	Spun (*n* = 23)	Both (*n* = 45)
No. of OTUs						
Mean (SD)	237.5 (122.4)	243.5 (145.1)	247.0 (137.7)	271.8 (271.8)	264.7 (235.2)	276.2 (214.0)
Median	251.0	224.0	247.5	247.0	217.5	230
Minimum-maximum	4–510	7–606	4–619	10–639	6–1,016	6–1,016
Chao1 estimator						
Mean (SD)	310.2 (147.7)	317.0 (159.7)	333.3 (164.4)	341.6 (205.0)	336.9 (253.0)	362.5 (255.9)
Median	340.9	283.4	334.2	315.1	295.6	321.1
Minimum-maximum	7–570.5	7.2–706.0	7–740.6	20.5–780.2	7.5–1,085.7	7.5–1,193.4
Shannon’s index						
Mean (SD)	1.6 (0.8)	1.7 (0.9)	1.7 (0.9)	1.8 (0.8)	1.7 (0.8)	1.7 (0.8)
Median	1.5	1.8	1.7	1.9	1.7	1.8
Minimum-maximum	0.3–3.3	0.5–3.3	0.3–3.3	0.4–3.2	0.3–3.4	0.3–3.4
Inverse Simpson’s index						
Mean (SD)	3.4 (2.8)	4.0 (3.2)	3.7 (3.0)	3.9 (2.6)	3.9 (3.5)	3.9 (3.0)
Median	2.6	3.0	2.7	3.3	2.5	3.1
Minimum-maximum	1.1–14.6	1.2–13.2	1.1–14.6	1.1–11.6	1.1–17.0	1.1–17.0

Overall, our analyses show a clear difference between patients with LUTS and controls, namely, that the *Enterobacteriaceae* was the most abundant taxon associated with disease and that *Streptococcus* was the most abundant taxon associated with health. However, it is clear that the method of sample processing enriches for different taxa. Specifically, centrifugation enriched *Enterococcus* in patient samples and *Staphylococcus* in control samples.

### Comparison of sediment culture and sequencing approaches.

**FIG 5:**
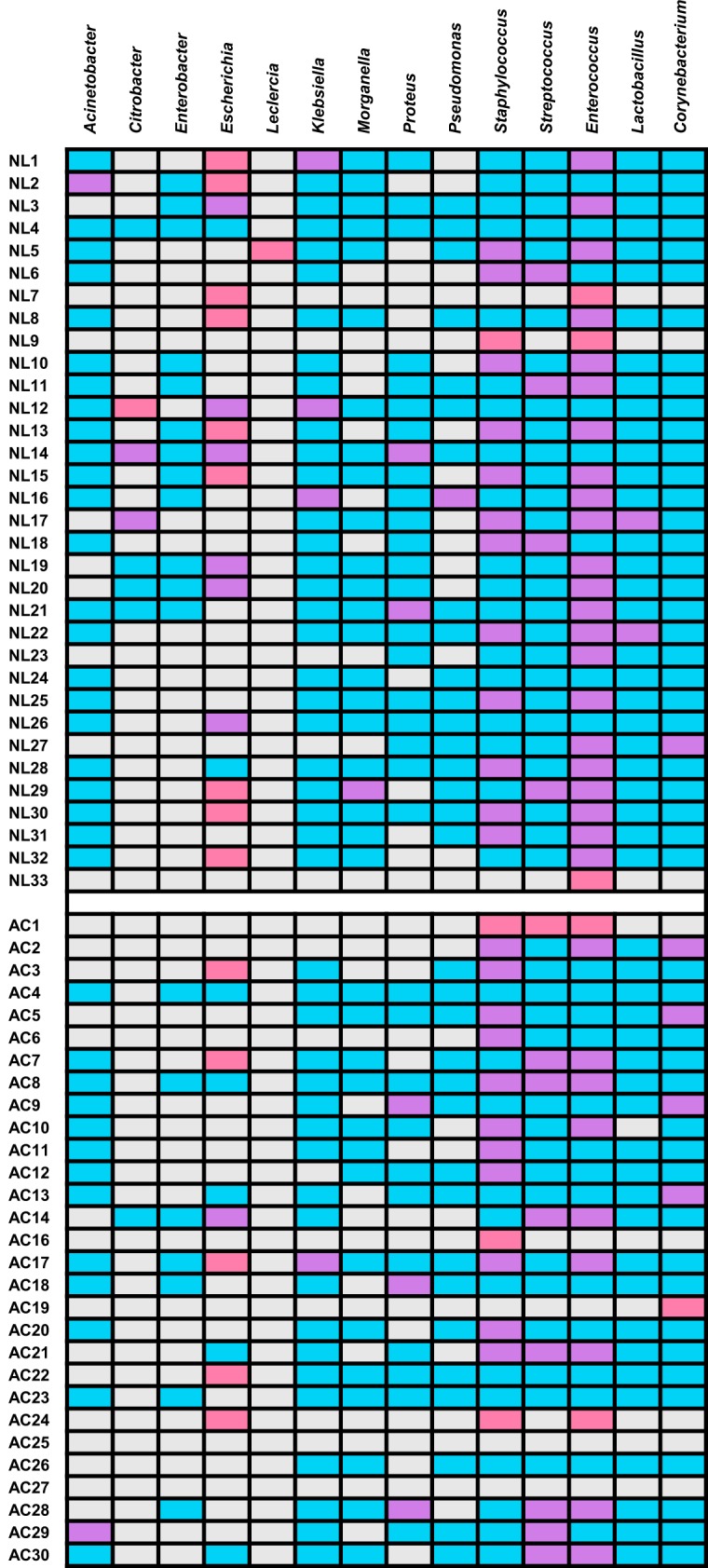
Comparison of genus-level taxa detected by sediment culture and 16S rRNA gene sequencing for new patient (NL) spun samples (*n* = 33) and asymptomatic control (AC) spun samples (*n* = 29). Interpretations are as follows: pink, bacteria identified by culture only; blue, bacteria identified by sequencing only; purple, bacteria identified by both culture and sequencing; cream, bacteria identified by neither culture nor sequencing.

As shown in Fig. 5, color-coded matrices consisting of all cultured bacterial taxa that were detected by centrifuged sediment culture and 16S rRNA gene sequencing were generated for new patient centrifuged samples and control centrifuged urine samples. Among all centrifuged samples, 37 (59.7%) of 62 contained bacteria that were detected by both culture and sequencing methods. Three (9.1%) patient and four (13.8%) control centrifuged samples were sequence negative but culture positive. Two (6.1%) patient and three (10.3%) centrifuged control samples were culture negative but sequence positive. *Enterococcus* was the most frequently identified bacterium from both the sediment culture and sequencing methods. This genus was identified from 22 (66.7%) new patient samples. *Staphylococcus* was the most frequently identified bacterium by both methods from control samples. This genus was identified from 11 (37.9%) centrifuged control samples. *Escherichia* demonstrated the most cases (*n* = 14) in which it was detected by sediment culture but remained undetected by sequencing. One new patient sample (NL5) grew *Leclercia* on sediment culture, but this particular taxon was not detected by sequencing. This analysis shows that the sequencing method is vastly superior to culture techniques for bacterial detection and that culture methods do not recover a large number of bacterial species, including recognized uropathogens.

## DISCUSSION

A recent commentary in the *Journal of Clinical Microbiology* emphasized how the diagnostic landscape for UTI is becoming increasingly challenging in the face of alternatives to standard culture (53). The primary purpose of this study was to evaluate the routine MSU culture performed in UK diagnostic laboratories. This evaluation is crucial, since culture is considered the gold standard diagnostic test for confirming or excluding UTI in symptomatic patients, although evidence suggests that it may be deficient (6, 7). In the United Kingdom, symptom-based empirical antibiotic treatment for acute UTI is recommended (54). The evidence suggests that the diagnostic accuracy of acute symptoms in previously asymptomatic subjects outperforms routine culture-based diagnosis (55). In contrast, relapsing patients with LUTS are not as straightforward and may demonstrate a distinct microbiota under the influence of prescribed antimicrobials. The cohort of relapsing patients was consequently excluded from genomic analysis to aid in clarifying the differences in urinary microbial communities between first-visit patients with LUTS and health. Despite testing negative on routine MSU culture, such patients have been shown to harbor uropathogens when their urine is examined with more sensitive methods (13, 28, 29, 36, 42). Furthermore, symptomatic resolution has been observed following antibiotic treatment in this type of patient (56). Therefore, we questioned whether current routine MSU cultures with a >10^5^-CFU/ml threshold were sensitive enough to detect UTI in patients presenting with LUTS.

Following a symptomatic assessment of LUTS patients, we scrutinized the microbial composition of each patient and control MSU culture and compared these results to the results reported by the hospital diagnostic laboratory, as well as to the organisms identified using other alternative culture and molecular methods. Crucially, we found that the routine MSU culture results reported by the diagnostic laboratory failed to discriminate between the patient and control groups. Less than 10% of patients with a clinically suspected UTI had a positive MSU culture, with only 4 of 33 new patient cultures producing monomicrobial growth at 10^5^ CFU/ml. In contrast, bacterial DNA sequencing revealed bacteria, including recognized uropathogens, in 32 new patients, including symptomatic patients who were reported to have a negative MSU culture. In this study, 16S rRNA gene sequencing was not applied as a diagnostic test, but rather, it was used to determine whether recognized uropathogens were missed with routine culture techniques and to characterize symptomatic and healthy urinary bacterial communities.

Our second key finding was that MSU culture grossly underestimated the amount of polymicrobial growth in patient urine. For example, 36.4% of new patient urine samples grew mixed growths; this number rose to 63.6% when more uncentrifuged urine was plated and to 84.8% in the case of centrifuged sediment cultures. Although it is now recognized that even healthy urine contains bacteria (41), which certainly complicates the diagnostic landscape, our enhanced culture and genomic analyses showed a clear difference between the taxa isolated from patients and those isolated from controls. Specifically, *Enterobacteriaceae* was the dominant family in new patients, whereas *Streptococcus* was foremost in the asymptomatic controls. These findings, taken together with other reports, suggest that automatically discounting polymicrobial cultures may dismiss symptomatic patients in need of treatment.

There is, of course, a formal possibility that the mixed growths reported here could simply be the result of contamination by skin bacteria during sample collection. In the United Kingdom, this possibility is addressed by the UK Standards for Microbiology Investigations, which recommends the counting of squamous epithelial cells in the urine as a marker for contamination. However, their utility for this purpose is complicated by the fact that exfoliation of urothelial cells as an innate immune response to UTI is a recognized phenomenon which is widely used as a surrogate marker of infection in both mice and humans (14, 21, 57–65). Indeed, using antibodies against uroplakin III, a highly specific marker for cells originating from the urinary tract, we previously demonstrated that the majority of epithelial cells shed in the urine of symptomatic patients originate from the bladder (14). Finally, were the presence of mixed growths merely the result of contamination, we would not expect their presence to cluster statistically with symptoms, as they did in this study.

Of note, some UTI-associated organisms were shared at the culture level by symptomatic and asymptomatic patients: *Corynebacterium*, *Enterococcus*, *Escherichia*, *Klebsiella*, *Proteus*, *Staphylococcus*, and *Streptococcus*. The presence of uropathogenic species in controls could reflect a lack of the appropriate virulence factor expression (11) or immune differences in the host (e.g., see reference 66). However, similar community profiles in the bladder have been seen by others (13, 28, 29, 42, 67), which makes it likely that the ability to be pathogenic is highly context dependent and could therefore be influenced by the presence of other species. Such pathobiont relationships are common in the microbial world (68) and in the case of UTI have been reported in mixed infections with E. coli and *Enterococcus* (25, 26). Furthermore, voided urine passes through the urethra as well as the bladder; the microbial ecology of the urethra is poorly understood in the case of UTI (69–71), and further studies are needed to understand what role such urethral communities might play.

Further afield, microbial community inhabitants in adjacent niches, such as the vagina and perianal region, may also influence what species induce UTI pathophysiology. For example, recent work by Gilbert et al. showed that transient exposure to the vaginal organism Gardnerella vaginalis could induce dysbiosis, reactivating dormant E. coli in previously infected mouse bladders (64). Other interactions could be protective; for example, the vaginal commensal Lactobacillus crispatus, which is known to make the vagina less hospitable to certain Gram-negative perianal uropathogens, may decrease the instance of recurrent UTI (72, 73). Indeed, our results showing that *Lactobacillus* is a dominant organism among the control samples, consistent with reports from other groups comparing the urine of patients with LUTS with that of asymptomatic controls, support this notion (28). Further research, including detailed sequence comparisons, is required to understand the mobility and interspecies effects of the various microbial inhabitants in the perianal region and the urethra region. This could further our understanding of the significance of polymicrobial communities in UTI.

Of interest, the process of centrifugation had a major effect on which taxa dominated, enriching *Enterococcus* in patient samples and *Staphylococcus* in controls. This is perhaps unsurprising, given that uropathogens are known to strongly adhere to and, in some cases, invade urothelial cells (14). These cells are shed into the urine via an innate immune response that is yet to be fully characterized (63, 74). Therefore, a significant proportion of bacteria in such specimens—and perhaps those most relevant for pathophysiology—could be cell associated and not planktonic. The MSU culture samples a small amount of urine supernatant and does not access the cell-rich sediment that settles by gravity. Several reports from the Brubaker group also recognized the limitations of standard culture and developed the expanded quantitative urine culture (EQUC) protocol. This involves inoculating uncentrifuged urine onto a broader selection of culture media for optimal species isolation (28, 36, 42). The approach demonstrated that the routinely performed standard quantitative culture missed 67% of recognized uropathogens, with the authors suggesting that the method be used to supplement the standard culture (42). They did not, however, analyze centrifuged sediment with this protocol.

In addition to factors beyond control, such as the volume of fluid intake before urine sampling, even the use of a broader selection of culture media has its limitations (31). The cultured urinary community depends on the types of specimens analyzed, the techniques employed, the nutrient medium used, the laboratory conditions (e.g., pH, temperature, O_2_ concentration), as well as the viability and facultative and fastidious natures of urinary tract organisms. Such factors can distort the species richness of the urinary tract community. Additionally, bacteria may exist in the viable but not cultivable (VBNC) state, also referred to as conditionally viable environmental (CVEC) cells, active but not cultivable (ABNC) cells, or dormant cells (75). Such species are more reliably represented by nucleic acid-based approaches.

In their studies, Brubaker and colleagues examined bladder urine specimens (specifically collected by a transurethral catheter [CSU] or suprapubic aspiration [SPA]) (28, 33, 36, 42). In our study, we used a clean-catch MSU specimen collection technique for several reasons. The aim of this study was to evaluate the routine MSU culture technique, which is the primary method in the clinic for collecting a urine sample for culture. In symptomatic patients, catheterization is potentially an invasive, uncomfortable, and painful method of collection that has also been reported to increase the risk of developing an infection (76). Furthermore, catheterization may bypass organisms colonizing the urethra, which may represent an early-stage UTI or contribute to the infection. Hooton et al. discussed this as a possible reason for why a lower threshold is usually applied to CSU cultures and a higher count is considered for MSU cultures, since it is unclear if the latter collection method has sampled bacteria from the bladder and/or the urethral region (27).

Female subjects formed the majority of patients and controls, in line with the demographics of UTI. Given that a previous study found some differences between the microbial ecology in male and female bladders (77), it would be interesting to further explore the effect of sex on symptomatic urinary microbiota.

The lack of a statistical difference between patient and control uncentrifuged samples was consistent with the findings of other studies (28, 78) and could be attributed to the small study cohort. However, statistical comparison of all patient samples (uncentrifuged and centrifuged) to all control samples (uncentrifuged and centrifuged) using the nonparametric Kruskal-Wallis test did confirm a significantly higher median number of OTUs in patients than in controls. This suggests that incorporating centrifuged urine samples may clarify the distinction between patient and control bacterial community richness.

While region-specific 16S rRNA gene sequencing using the MiSeq platform provided a less biased representation of the urinary bacterial community, it is important to acknowledge the limitations of this method. Due to the short-read sequencing approach used, approximately 30% (approximately 500 nucleotides) of the 16S rRNA gene was amplified, which makes identification at the genus level possible but reliable taxonomic assignment of reads at the species level elusive. Our comparison of taxa identified by culture and 16S rRNA gene sequencing revealed that the sequencing method was more capable than the centrifuged sediment culture technique of identifying the cultivable bacterial taxa from patient and control samples. However, the sediment culture method permitted bacterial identification at the species level, which could not be achieved reliably by 16S rRNA gene sequencing directly from urine. Of note, *Escherichia* demonstrated one of the highest rates of detection by culture but was often undetected by DNA sequencing among centrifuged and uncentrifuged samples. This genus belongs to the *Enterobacteriaceae* family, the members of which are reported to be challenging to identify reliably at the genus level due to the high degree of sequence similarities (79). Given that E. coli is a prominent uropathogen, improvements are needed. A molecular species-level characterization could be achieved using the more recently developed third-generation sequencing technologies, including the MinION platform series (80). The potential to reconstruct more than 90% of the 16S rRNA gene makes species-level characterization possible.

This study underlines the potential weaknesses of the MSU culture for diagnosing UTI in patients affected by LUTS, which should now be assessed further in a larger, multicenter study. These patients appear to harbor chronic infection, missed by quantitative cultures and most effectively demonstrated by nonculture techniques. These methods shed light on the complexity of the bacterial communities within these patients, whose symptoms and urinary white blood cell count may be the best indicators of infection until the disease landscape is better understood. In the meantime, those responsible for UTI detection, diagnosis, and patient care, including clinicians and microbiologists, may wish to use caution when interpreting a negative or mixed-growth MSU culture result in symptomatic patients, as well as reassess reporting and treatment guidelines to arrive at a solution most appropriate for patients in a way that also preserves antibiotic stewardship efforts (53). Ultimately, a point-of-care test based on a sound understanding of the relevant microbiomes using emerging portable genomic technology would revolutionize the diagnostic landscape for this common affliction.

## Supplementary Material

Supplemental file 1
