# Supplementary material for: Reassessment of Routine Midstream Culture in Diagnosis of Urinary Tract Infection
Source: J Clin Microbiol. 2019 Feb 27;57(3):e01452-18. doi: 10.1128/JCM.01452-18 (PMC6425166; doi:10.1128/JCM.01452-18)
Supplement: Supplemental file 1 [file JCM.01452-18-s0001.pdf]

SUPPLEMENTARY FIGURE LEGENDS AND TABLES

Table S1. List of primary and secondary 16S rRNAV5-V7 primers used.

Figure S1. Stacked plots of microbial taxa identified at the family and genus levels identified for three mock communities (Mock 1-3). From left to right: Mock 3 = HM-782D (Bei Resources, USA), Mock 1 = customised community of 10 bacterial species at 10^{10}-10^{11} cfu/ml and Mock 2 = customised community of 10 bacterial species in equimolar DNA concentrations (21.75 ng/µl).

Table S2. Frequencies of individual LUTS for new patients and treatment patients.

Figure S2. Percentage frequencies of organisms identified from the polymicrobial midstream urine cultures that were performed in UCL's research laboratories for new patients (A, *n* = 7), relapsed patients (B, *n* = 4), and controls (C, *n* = 5).

Figure S3. Microbial species identified on new patient (blue), control (magenta) and relapse patient (green) sediment cultures. A = *Enterobacteriaceae* members and other non-enteric bacilli, B = *Staphylococcus*, C = *Enterococcus* and *Streptococcus* and D = *Corynebacterium* and other organisms.

Table S3. Hierarchical classifications of bacterial DNA sequences identified from new patients and controls urine specimens.

PCR PRIMER	SEQUENCE 5’to 3’
**Primary**	
785For	GGATTAGATACCCBRGTAGTC
1175Rev	ACGTCRTCCCCDCCTTCCTC
**Secondary Forward**	
P5_FWD01	AATGATACGGCGACCACCGAGATCTACAC**TAGATCGC**ACGTACGTACGTGGATTAGATACCCBRGTAGTC
P5_FWD02	AATGATACGGCGACCACCGAGATCTACAC**CTCTCTAT**ACGTACGTACGTGGATTAGATACCCBRGTAGTC
P5_FWD03	AATGATACGGCGACCACCGAGATCTACAC**TATCCTCT**ACGTACTGACGTGGATTAGATACCCBRGTAGTC
P5_FWD04	AATGATACGGCGACCACCGAGATCTACAC**AGAGTAGA**ACGTACGTACGTGGATTAGATACCCBRGTAGTC
P5_FWD05	AATGATACGGCGACCACCGAGATCTACAC**GTAAGGAG**ACGTACGTACGTGGATTAGATACCCBRGTAGTC
P5_FWD06	AATGATACGGCGACCACCGAGATCTACAC**ACTGCATA**ACGTACGTACGTGGATTAGATACCCBRGTAGTC
P5_FWD07	AATGATACGGCGACCACCGAGATCTACAC**AAGGAGTA**ACGTACGTACGTGGATTAGATACCCBRGTAGTC
P5_FWD08	AATGATACGGCGACCACCGAGATCTACAC**CTAAGCCT**ACGTACGTACGTGGATTAGATACCCBRGTAGTC
**Secondary Reverse**	
P7_REV01	CAAGCAGAAGACGGCATACGAGAT**TCGCCTTA**AGTCAGTCAGCCACGTCRTCCCCDCCTTCCTC
P7_REV02	CAAGCAGAAGACGGCATACGAGAT**CTAGTACG**AGTCAGTCAGCCACGTCRTCCCCDCCTTCCTC
P7_REV03	CAAGCAGAAGACGGCATACGAGAT**TTCTGCCT**AGTCAGTCAGCCACGTCRTCCCCDCCTTCCTC
P7_REV04	CAAGCAGAAGACGGCATACGAGAT**GCTCAGGA**AGTCAGTCAGCCACGTCRTCCCCDCCTTCCTC
P7_REV05	CAAGCAGAAGACGGCATACGAGAT**AGGAGTCC**AGTCAGTCACCCACGTCRTCCCCDCCTTCCTC
P7_REV06	CAAGCAGAAGACGGCATACGAGAT**CATGCCTA**AGTCAGTCAGCCACGTCRTCCCCDCCTTCCTC
P7_REV07	CAAGCAGAAGACGGCATACGAGAT**GTAGAGAG**AGTCAGTCAGCCACGTCRTCCCDCCTTCCTC
P7_REV08	CAAGCAGAAGACGGCATACGAGAT**CCTCTCTG**AGTCAGTCAGCCACGTCRTCCCCDCCTTCCTC
P7_REV09	CAAGCAGAAGACGGCATACGAGAT**AGCGTAGC**AGTCAGTCAGCCACGTCRTCCCCDCCTTCCTC
P7_REV10	CAAGCAGAAGACGGCATACGAGAT**CAGCCTCG**AGTCAGTCAGCCACGTCRTCCCCDCCTTCCTC
P7_REV11	CAAGCAGAAGACGGCATACGAGAT**TGCCTCTT**AGTCAGTCAGCCACGTCRTCCCCDCCTTCCTC
P7_REV12	CAAGCAGAAGACGGCATACGAGAT**TCCTCTAC**AGTCAGTCAGCCACGTCRTCCCCDCCTTCCTC

Table S1. List of primary and secondary 16S rRNAV5-V7 primers used.

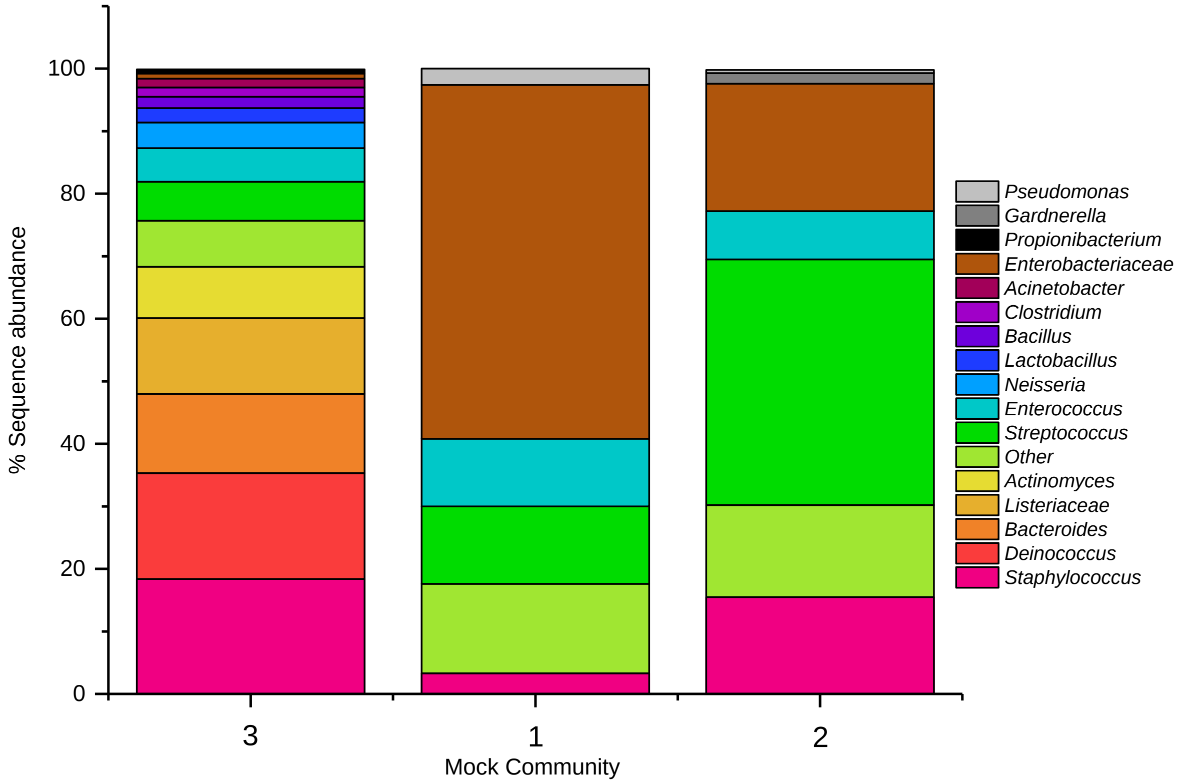

Figure S1. Stacked plots of microbial taxa identified at the family and genus levels identified for three mock communities (Mock 1-3). From left to right: Mock 3 = HM-782D (Bei Resources, USA), Mock 1 = customised community of 10 bacterial species at 10^{10}-10^{11} cfu/ml and Mock 2 = customised community of 10 bacterial species in equimolar DNA concentrations (21.75 ng/µl).

SYMPTOM	FREQUENCY (%)		FISHER'S TEST χ^2	*P*-VALUE
	New patients (*n* = 33)	Relapsed patients (*n* = 30)		
**STRESS URINARY INCONTINENCE**				
**Cough/sneeze incontinence**	**13 (39.4)**	**2 (6.7)**	**9.3**	**< 0.05**
Exercise incontinence	5 (15.2)	1 (3.3)	2.5	0.199
Lifting incontinence	0 (0.4)	1 (0.3)	1.1	0.476
Laughing incontinence	3 (9.1)	1 (3.3)	0.9	0.614
Positional incontinence	0 (0.0)	1 (3.3)	1.1	0.476
Coughing with urgency incontinence	3 (9.1)	2 (6.7)	0.1	1.000
**URGENCY URINARY INCONTINENCE**				
**Urgency**	**22 (66.7)**	**11 (36.7)**	**5.7**	**< 0.05**
Urgency incontinence	11 (33.3)	4 (13.3)	3.5	0.080
Cold weather urgency	10 (30.3)	3 (10.0)	4.0	0.064
Running water urgency	9 (27.3)	3 (10.0)	3.0	0.112
Running water incontinence	1 (3.0)	1 (3.3)	0.01	1.000
**Latchkey urgency**	**12 (36.4)**	**3 (10.0)**	**6.0**	**< 0.05**
**Latchkey incontinence**	**7 (21.2)**	**0 (0.0)**	**7.2**	**< 0.05**
**Waking up urgency**	**17 (51.5)**	**6 (20.0)**	**6.7**	**< 0.05**
Waking rising incontinence	3 (9.1)	4 (13.3)	0.3	0.700
**Anxiety urgency**	**15 (45.5)**	**3 (10.0)**	**9.7**	**< 0.05**
Premenstrual aggravation*	5 (15.2)	4 (13.3)	0.01	1.000
**VOIDING**				
Hesitancy	18 (54.5)	17 (56.7)	0.02	1.000
Reduced stream	21 (63.6)	19 (63.3)	0.001	1.000
Intermittent stream	14 (42.4)	16 (53.3)	0.8	0.454
Straining to void	17 (51.5)	13 (43.3)	0.4	0.616
**Terminal dribbling**	**23 (69.7)**	**12 (40.0)**	**5.6**	**< 0.05**
Post-void dribbling	9 (27.3)	7 (23.3)	0.1	0.778
Double voiding	15 (45.5)	14 (46.7)	0.01	1.000
Incomplete emptying	18 (54.5)	11 (36.7)	2.0	0.208
**PAIN**				
Filling bladder pain	18 (54.5)	15 (50.0)	0.1	0.803
Bladder pain fully relieved by voiding	3 (9.1)	3 (10.0)	0.02	1.000
Bladder pain partially relieved by voiding	14 (42.4)	10 (33.3)	0.6	0.604
Bladder pain unrelieved by voiding	0 (0.0)	1 (3.3)	1.1	0.476
Bladder or suprapubic pain	16 (48.5)	20 (66.7)	2.1	0.203
Loin pain	8 (24.2)	12 (40.0)	1.8	0.278
Dysuria	6 (18.2)	8 (26.7)	0.7	0.547
Urethral pain	7 (21.2)	13 (43.3)	3.5	0.103
Genital pain	4 (12.1)	4 (13.3)	0.02	1.000
Iliac fossa pain	10 (30.3)	8 (26.7)	0.1	0.787
Radiating leg pain	4 (12.1)	4 (13.3)	0.02	1.000
Pain during void	4 (12.1)	8 (26.7)	2.2	0.202
Post-void bladder pain	8 (24.2)	3 (10.0)	2.2	0.189

Table S2. Frequencies of individual LUTS for new patients and treatment patients.

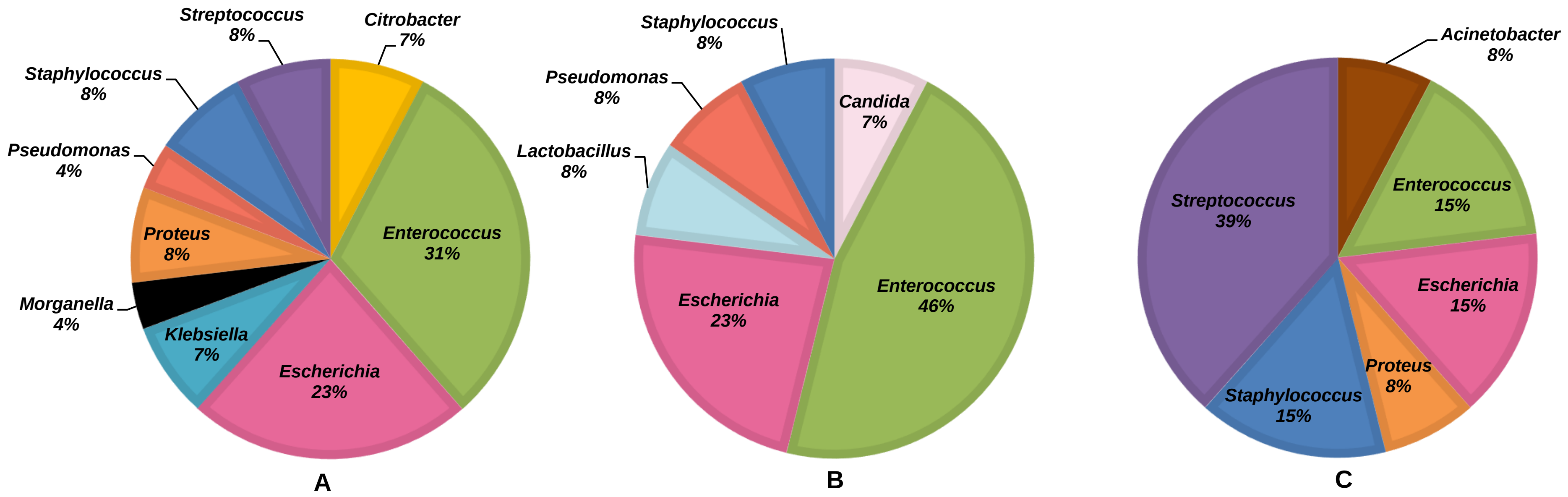

Figure S2. Percentage frequencies of organisms identified from the polymicrobial midstream urine cultures that were performed in UCL's research laboratories for new patients (A, n = 7), relapsed patients (B, n = 4), and controls (C, n = 5).

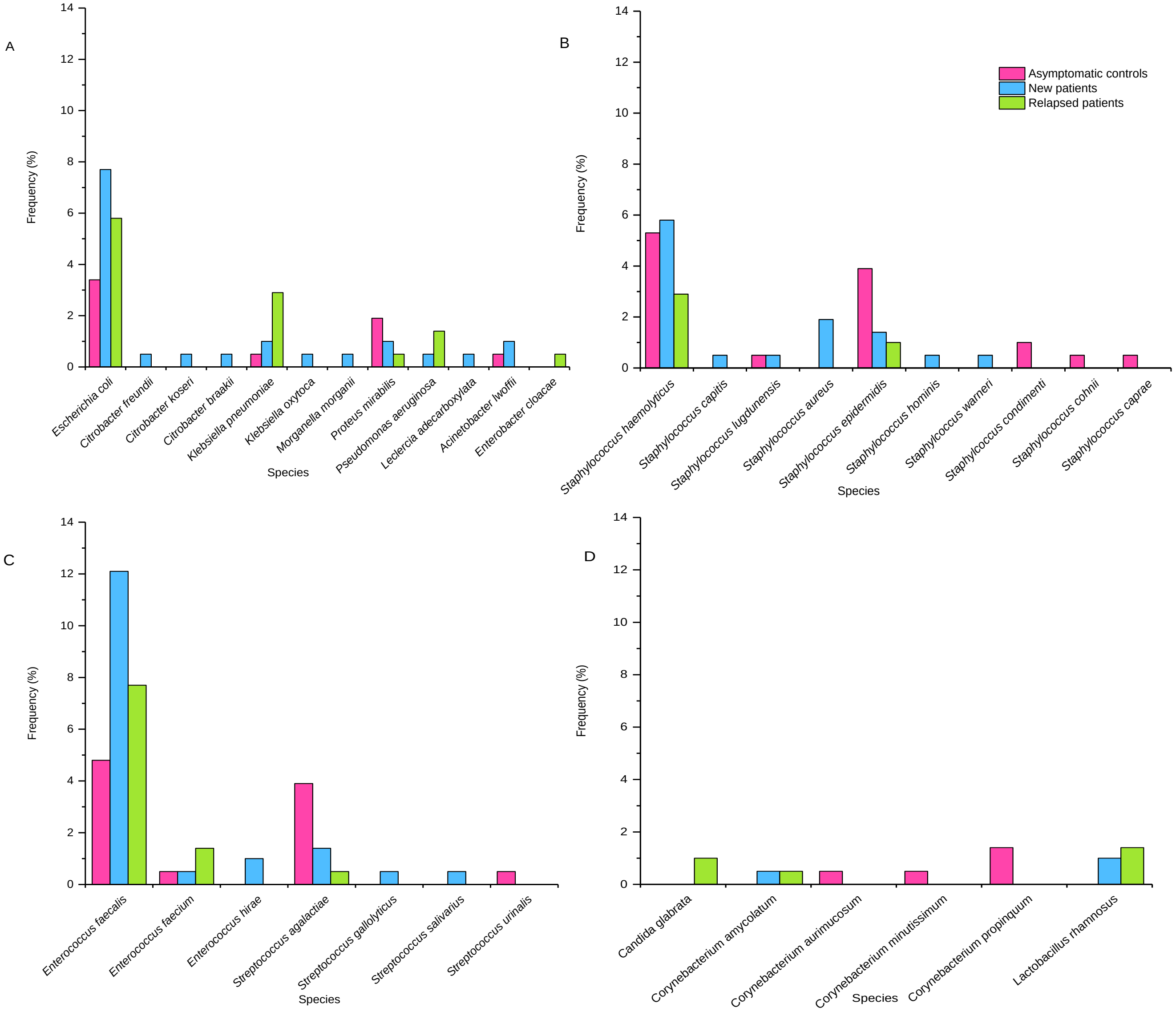

Figure S3. Microbial species identified on new patient (blue), control (magenta) and relapse patient (green) sediment cultures. A = *Enterobacteriaceae* members and other non-enteric bacilli, B = *Staphylococcus*, C = *Enterococcus* and *Streptococcus* and D = *Corynebacterium* and other organisms.

SEQUENCE CLASSIFICATION	CONTROLS	NEW PATIENTS
**Number**		
Phylum	18	20
Class	36	44
Order	69	71
Family	145	146
Genus	324	343
**Phyla sub-classifications (Median sequence abundance [%])***		
Firmicutes	66.2	41.0
Actinobacteria	6.2	6.4
Proteobacteria	1.6	4.2
Bacteroidetes	0.5	0.2

Table S3. Hierarchical classifications of bacterial DNA sequences identified from new patients and controls urine specimens.

Footnotes:

* Median sequence abundance of phyla sub-classifications with a sequence abundance of > 0.1%.
